# The Development and Implementation of a Model to Facilitate Self-Awareness of Professionalism for Enrolled Nurses

**DOI:** 10.1177/08980101221134758

**Published:** 2022-11-08

**Authors:** Tracey de Klerk, Annie Temane, Charlene Downing

**Affiliations:** 61799 University of Johannesburg, South Africa

**Keywords:** facilitation, self-awareness, professionalism, enrolled nurses, model development, model implementation

## Abstract

Within the South African context, the nursing profession comprises different nursing cadres. The enrolled nurse is considered a sub-category of nursing and therefore does not carry the title of ‘professional’ as in a professional nurse. The purpose of the study was to develop, describe, implement, and evaluate a model for the facilitation of self-awareness for the professionalism of enrolled nurses at a specific nursing agency in Gauteng. A theory generating, qualitative, exploratory, descriptive and contextual design was used and was conducted following Chinn and Kramer's four stages of model development. The model can benefit nursing education because it relates to an essential aspect of growth and maturity in one's career. Ultimately, the facilitation of self-awareness for professionalism can advance one's career, or the lack of self-awareness may impede one's career. Developing, describing, implementing and evaluating this model to facilitate self-awareness for the professionalism of enrolled nurses at a specific nursing agency in Gauteng provides an original contribution to the theory in nursing professionalism and ethos. This model can be utilised as a tool to facilitate self-awareness for the professionalism of enrolled nurses at a nursing agency.

## What is Known?

- A nurse's portrayed professionalism influences a patient's perception of the quality of care rendered.
- Nurses who ensure that their appearance and behaviour is exemplary are perceived as professional.
- Research studies concerning enrolled nurses’ self-awareness of professionalism, in South Africa, are non-existent.
- The transformation in nursing education, in South Africa, has impacted the enrolled nurse's training programme resulting in a cessation in the enrolled nurse's training programme.
- 78 652 enrolled nurses are still registered with the South African Nursing Council.

## What is new?

- This model facilitates self-awareness for professionalism of enrolled nurses, who are working through a nursing agency, in Gauteng.
- The development of the model to facilitate self-awareness for professionalism of enrolled nurses
- The evaluation of the model to facilitate self-awareness for professionalism of enrolled nurses

## Introduction

This article presents the development and implementation of a model to facilitate self-awareness of professionalism for enrolled nurses, in a specific nursing agency in South Africa. The article highlights fundamental “building blocks” necessary for a nursing model's framework, developed through empiric knowledge based upon a systematic, scientific approach using creative processes.

Nursing is a professional discipline, focussing on the wholeness of a person's health and healing, based on the knowledge of nursing (Smith, 2020, p. 2). The knowledge of nursing comprises of theories, philosophies, empiric knowledge and practical wisdom (Kivunja, 2018, pp. 44–53). Theories are viewed as notions or ideas guiding thinking about nursing (Smith, 2020, p. 7), consisting of a set of defined concepts and relational statements (Gray, Grove & Sutherland, 2017, 75–102). Literature indicates that the words: theory and model, are used interchangeably due to their ambiguous explanations (Gray *et al*., 2017: 75–102). Gray *et al*. (2017: 75–102) explain that in a conceptual model, the concepts are the “basic building blocks of the framework” (Walker & Avant, 2019, p. 167), and a model explains the phenomenon, expresses assumptions and a philosophical state. The major reason for theory development is to improve nursing practice, referring to the experiences encountered by the patient, the nurse and others in the environment (Chinn et al., 2022, p. 295). Therefore, it can be viewed that if enrolled nurses portrayed professionalism within the workplace, their nursing practice would resonate with quality nursing care (Smith, 2020, p. 7).

## Contextual Insights Related to Nursing in South Africa

Several pertinent contextual aspects are relevant to this study, relating to the sub-category of nursing cadre and the transformation in nursing, resulting from of the democratic dispensation in South Africa, after 1994. South Africa's nursing cadre has consisted of three categories, namely the registered nurse, enrolled nurse, and auxiliary nurse. An enrolled nurse, in South Africa, is defined as a person who was enrolled as a pupil nurse, by an accredited nursing school under the South African Nursing Council (SANC), while undergoing a two-year nursing course under Regulation 2175 (SANC, Nursing Act 50 of 1978). Completion of the course will lead to enrolment as a nurse and are known as an enrolled nurse. The enrolled nurse is viewed as a subcategory of nursing, functioning under the direct and indirect supervision of the registered nurse (SANC Nursing Act 50 of 1978, SANC Regulation No. 2598; Regulation No. 1649). This means enrolled nurses’ actions and procedures must be planned and initiated by a registered nurse (SANC Regulation No. 2598).

The enrolled nurse forms part of the nursing cadre in South Africa, and although this category of nurse does not have the title of ‘professional’ (as in a professional nurse), they form part of a professional body, namely the South African Nursing Council (SANC Regulation No. 2598).

The transformation in nursing education ceased the enrolled nurse's training programme, with the Nursing Act (Act 33 of 2005) making provisions for the creation of new nursing categories. This involved the new development of scopes of practice and related regulations in preparation for a new Higher Education Qualifications Sub-Framework (HEQSF) for aligned nursing qualifications (National Department of Health No. 42380, 2019:7–10; Zwane & Mtshali, 2019, pp. 2–3). The transformation in nursing education was supported by several factors: one being that Nursing Educational Institutions (NEI) each having different settings, with different models of governance, and different student management systems (National Department of Health No. 42380, 2019:7; Zwane & Mtshali, 2019, pp. 2–3). This resulted in unstandardised nursing education. Furthermore, enrolled nurses exited their training programme at the same exit level as during schooling; the transformation placed nursing education qualifications within the requirements for post-schooling. Lastly, there was a general lack of nationally determined minimum entry requirements into enrolled nurse programmes, which led to an overproduction of enrolled nurses as reported by the National Policy on Nursing Education and Training (National Department of Health No. 42380, 2019:7), however, prior to the global Covid-19 pandemic, Solidarity, a South African trade union, claimed that healthcare is nearing a crisis due to nursing staff shortages (Brits, 2019, p. 1).

The phasing out of the old nursing programmes was initiated in 2015, and nurses who completed nursing programmes before the new qualifications are referred to as having ‘legacy’ nursing qualifications (National Department of Health No. 42380, 2019:7–10).

The enrolled nurses’ delegated duties often entail greater patient contact than that of a registered nurse (Janse van Rensburg et al., 2020, p. 2). This resulted in a higher enrolled nurse-to-patient contact than registered nurse-to-patient contact, meaning more enrolled nurses are on the ‘floor’ than registered nurses (Janse van Rensburg et al., 2020, p. 2; Malatji et al., 2017, pp. 325–332). According to the South African Nursing Council's (SANC) annual report (SANC, Annual Report: 2017) indicated 78 652 enrolled nurses and 144 914 registered nurses are on SANC's register, providing a ratio of enrolled nurses to registered nurses as 1:1,8.

Even though the enrolled nurse's training programme has ceased, 78 652 enrolled nurses are still registered with SANC (SANC, Annual Report: 2017) and who still deliver patient care. Based on the high number of enrolled nurses, on SANC's Annual Report, who have contact with patients and deliver patient care, it is imperative that the enrolled nurse is self-aware of their displayed professionalism within the workplace.

## Background of the Study

Nursing is held as an iconic profession, with varying ideologies on how this profession should be portrayed (Hunt, 2020, pp. 19–22; Wills et al., 2018, pp. 30–40). A nurse's portrayed professionalism is commonly associated with the degree of the quality of care rendered (Parizad et al., 2018, pp. 54–59; Westbrook et al., 2021, pp. 31–37). McKay (2019:, p. 1) considers professionalism as an individual's competency to display the expected behaviour necessary within a specific environment. This study was conducted in a specific nursing agency, in Gauteng Province, South Africa. A nursing agency is a temporary employment agency, also known as a labour broker, allowing nursing personnel to voluntarily register with that nursing agency, with the intention of working temporary shifts at a specific healthcare facility (Bowyer, 2019, pp. 1–2; South Africa, Ministry of Health, 2012/13–2016/17). This practice is also known as ‘moonlighting’.

It is noteworthy to mention that the researcher was previously employed (2007–2009) at a different nursing agency that had a national footprint in South Africa, meaning that nursing agency provided healthcare facilities throughout South Africa with supplemental nursing staff. During that time, it was uncommon that nursing agencies employed clinical preceptors. Furthermore, currently, there is no literature available to provide any indication of clinical preceptors working at a nursing agency.

The specific nursing agency employs clinical preceptors who conduct orientation programmes with the nursing personnel prior to the temporary placement within specific healthcare facilities; furthermore, the clinical preceptors provide supervision during clinical practice and facilitate the application of theory to practice. Enrolled nurses form part of the nursing personnel. The nursing agency received telephonic complaints from managers in private healthcare facilities stating that the enrolled nurses from the nursing agency displayed unprofessional behaviour. The central concept was identified from the researcher's master's study (De Klerk, 2016), that was conducted in a specific nursing agency, in Gauteng Province, South Africa.

## Study Design

**Figure 1.**
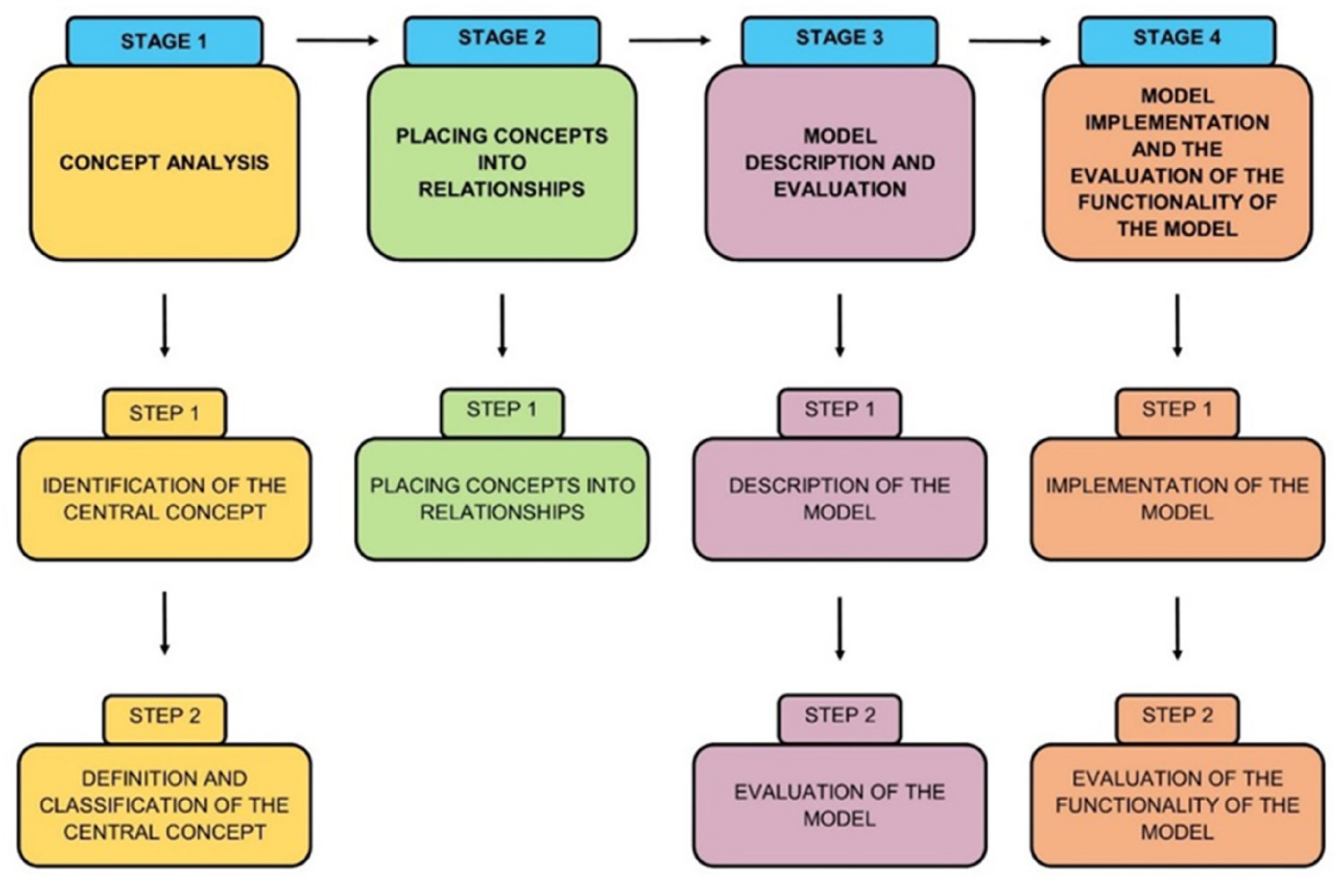
The four stages of model development and implementation.

A theory generative, qualitative, exploratory, descriptive and contextual research design was used. The researcher used four stages for generating the model (Chinn et al., 2022, pp. 148–157), namely: Stage 1: Concept analysis, Stage 2: Placing concepts into relationships, Stage 3: Model description and evaluation, and Stage 4: Model implementation and the evaluation of the functionality of the model. Each stage will be described. Walker and Avant's (2019:, pp. 167–184) guidance was used during concept analysis and placing concepts into relationships. Two models: Johari Model (Luft, 1969) and the Nursing Supervision Model (Severinsson, 2001), were integrated into the current model due to their unique theoretical assumptions. Figure 1 depicts the four stages followed in developing and implementing this model.

## Ethical Considerations

The ethical clearance was approved by the Research Ethics Committee (REC-01-61-2017), Higher Degree Committee (HDC-01-41-2017) and the CEO of the nursing agency. Participants read the information letter and signed the consent form before model implementation and data collection. The COVID-19 protocols were observed, face masks and hand sanitising occurred during the presentation of the model for implementation and during the unstructured interview.

## Developing and Implementing the Model

### Stage 1 - Concept Analysis

A concept analysis was performed according to Walker and Avant (2019:, pp. 167–184), with the purpose of examining the concept's structure and function. The process of concept analysis involved two steps, Step 1 – identifying the central concept, and Step 2 – defining and classifying the central concept.

#### Step 1: Identifying the Central Concept

**Figure 2.**
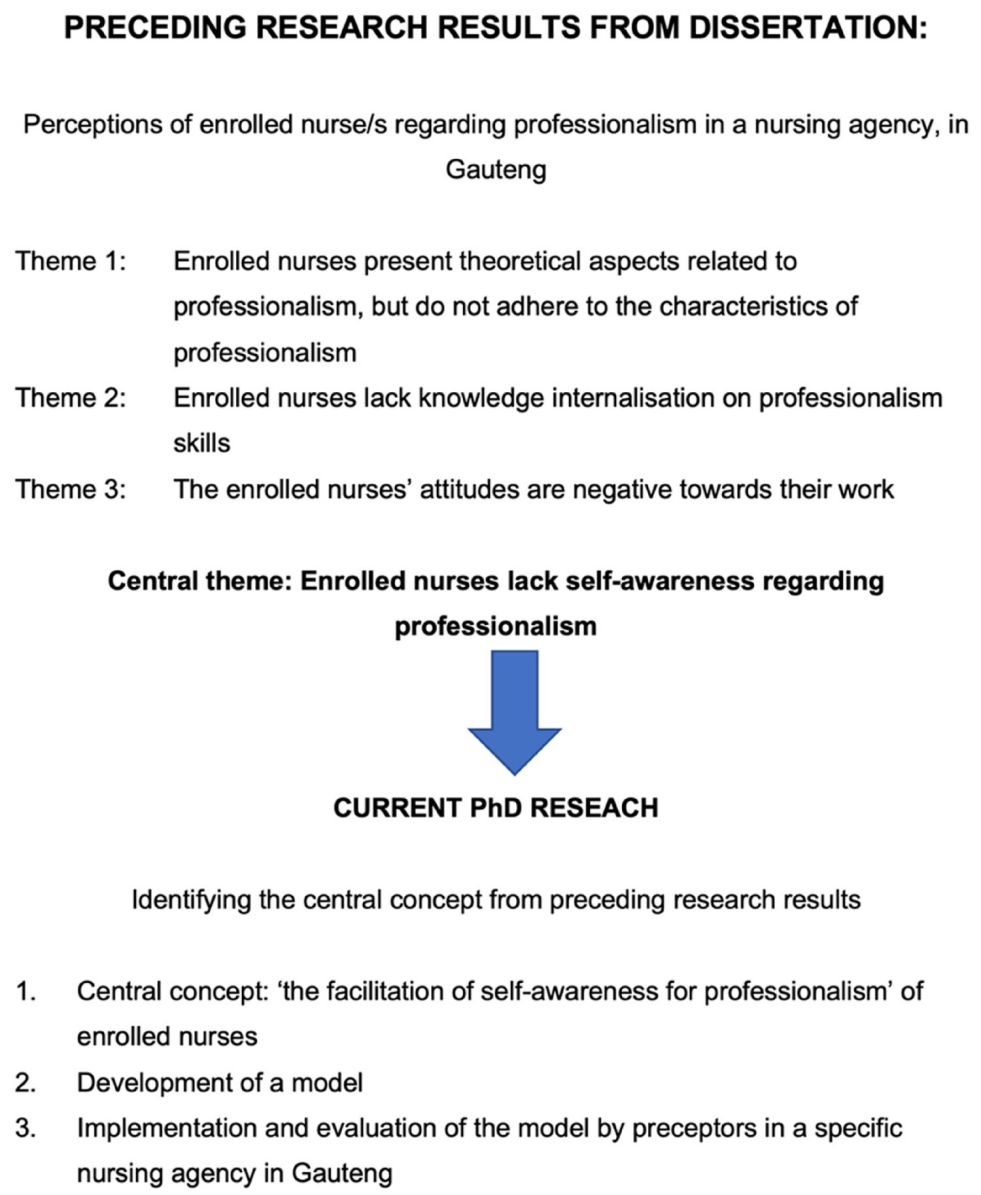
The reasoning map to identify the central concept.

The emerged themes from the researcher's previous dissertation were utilised as a frame of reference in identifying the central concept. Figure 2 depicts the reasoning map, indicating the sub-themes and central theme derived from the researcher's previous dissertation that led to the central theme in the researcher's study.

The central concept was identified as ‘the facilitation of self-awareness for professionalism’ for enrolled nurses.

#### Step 2: Defining and Classifying the Central Concept

Defining the central concepts involved an in-depth and broad search. During the literature search, the researcher noticed that there were contextual influences, meaning that there were aspects involved in the study that needed to be fully understood by the researcher and the reader to make sense of the implications that impacted the study.

#### Search Strategies

**Figure 3.**
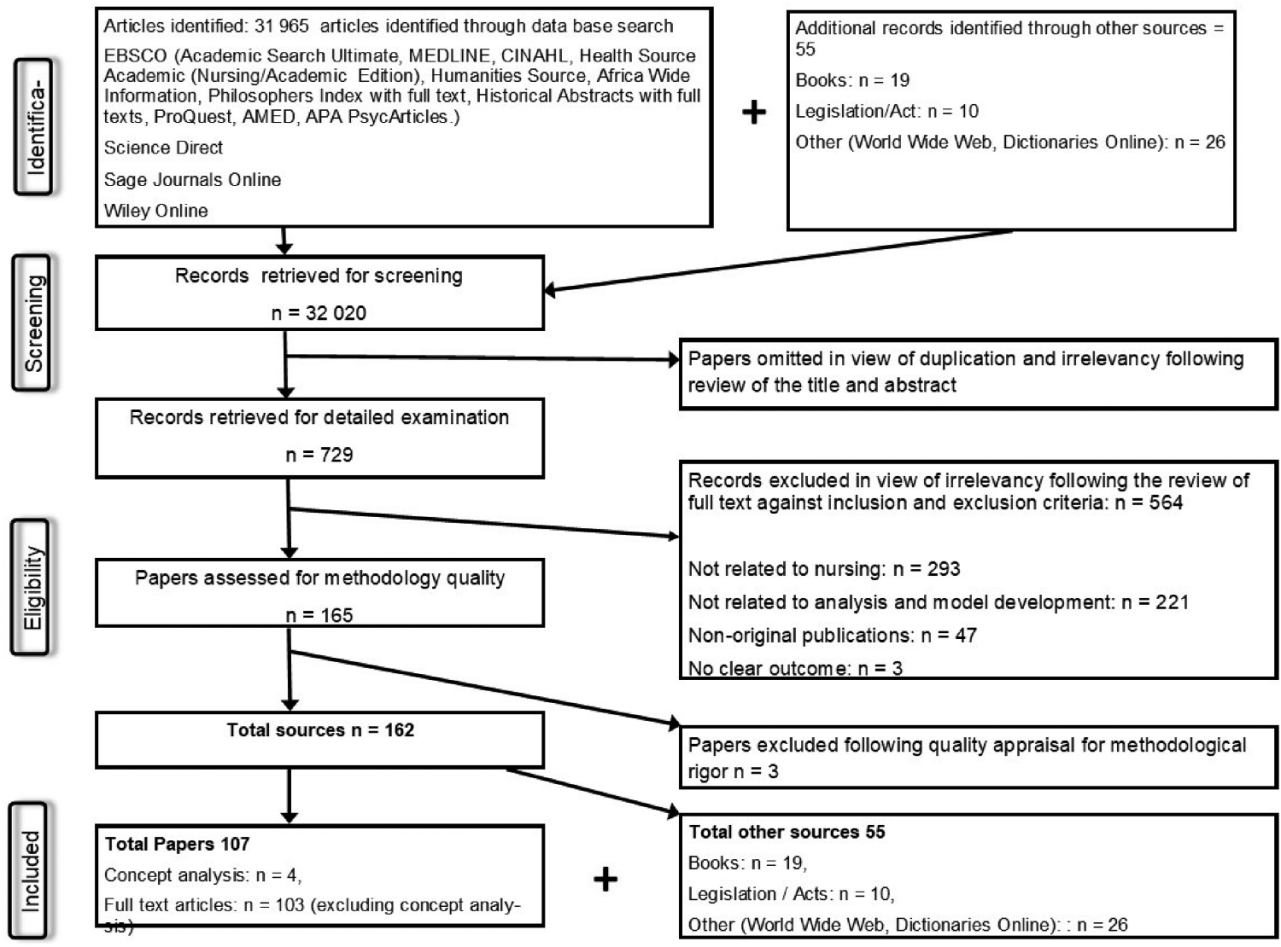
The Prisma.

The researcher consulted primary and secondary resources. Primary resources, that is the original source, at times were outdated as reference was made to 1985–2004. During the search, the researcher became aware of seminal work that explained certain concepts, such as professionalism, providing influential, contextualised and in-depth explanations relating to the South African nursing context. Seminal work included well-known theoretical frameworks, namely the Johari Model (Luft, 1969) and Nursing Supervision Model (Severinsson, 2001) that were pivotal to this study due to their influential development within the field of study. The researcher made use of the following databases for literature searches: Academic Search Ultimate, MEDLINE, CINAHL, Health Source (Nursing/Academic Edition), Humanities Source, Africa-Wide Information, Philosophers Index with Full Text, Historical Abstracts with Full Text, Religion and Philosophy Collection, ProQuest (Dissertations and Thesis) AMED and APA PsycArticles. The literature search continued in Sage Journals Online, Science Direct, Wiley Online, Google Books and the World Wide Web. These data bases were selected to ensure coverage of a broad spectrum of the phenomenon under study, including fields of psychology, sociology, education and nursing,

A Boolean search was conducted for each concept. Due to limited studies found, regarding the concept ‘professionalism’ and the enrolled nurse, the researcher undertook a literature synthesising strategy (Walker & Avant, 2019, p. 149, 152, 153). Literature synthesising strategies are encouraged by Walker and Avant (2019:, p. 127, 149) as a means to clarify concepts when they are unclear or unknown. Figure 3 depicts the Prisma.

#### Defined Concepts:

- Facilitation - ‘the clinical preceptor will provide guidance to enrolled nurses through a process of mobilising resources to facilitate self-awareness for professionalism’.
- Self-awareness – ‘The concept ‘self-awareness’ is defined as the enrolled nurse having self-knowledge and an internal locus of control’
- Professionalism - The concept of ‘professionalism’ for enrolled nurses within the context of this study can be defined as: ‘the enrolled nurse's competency to display behaviour that is expected within the environment’.

### Stage 2: Placing Concepts into Relationships

Relationship statements are statements that ascribe an association between two or more concepts (Chinn et al., 2022, pp. 153–154). These concepts, in this study, pertain to facilitation, self-awareness, professionalism, the enrolled nurse, and the clinical preceptor from the nursing agency.

#### The Relationship Statements:

- The clinical preceptor will operationalise the process by using dialogue to form a connection between themselves and the enrolled nurse, thereby enhancing a participatory approach to assist the enrolled nurse introspect on their own character traits and regulate their behaviour in their place of work.
- Through the mobilisation of resources, the enrolled nurse develops self-knowledge and an internal locus of control to display an image and demeanour at the expected level of performance in their environment.
- By introspecting on their own character traits, the enrolled nurse will ‘be aware of the self’, which will be a motivator to display proficiency in their conduct and appearance in the workplace.

### Stage 3: Model Description and Evaluation by Experts

A model is a symbolic representation of experience in words, pictorial or graphic diagrams (Chinn et al., 2022, pp. 169–176). A model development method was selected to reflect the phenomenon of the enrolled nurses within their work environment, namely the private healthcare facility and the nursing agency, and their development of self-awareness to portray professionalism.

#### Overview of Model Description

**Figure 4.**
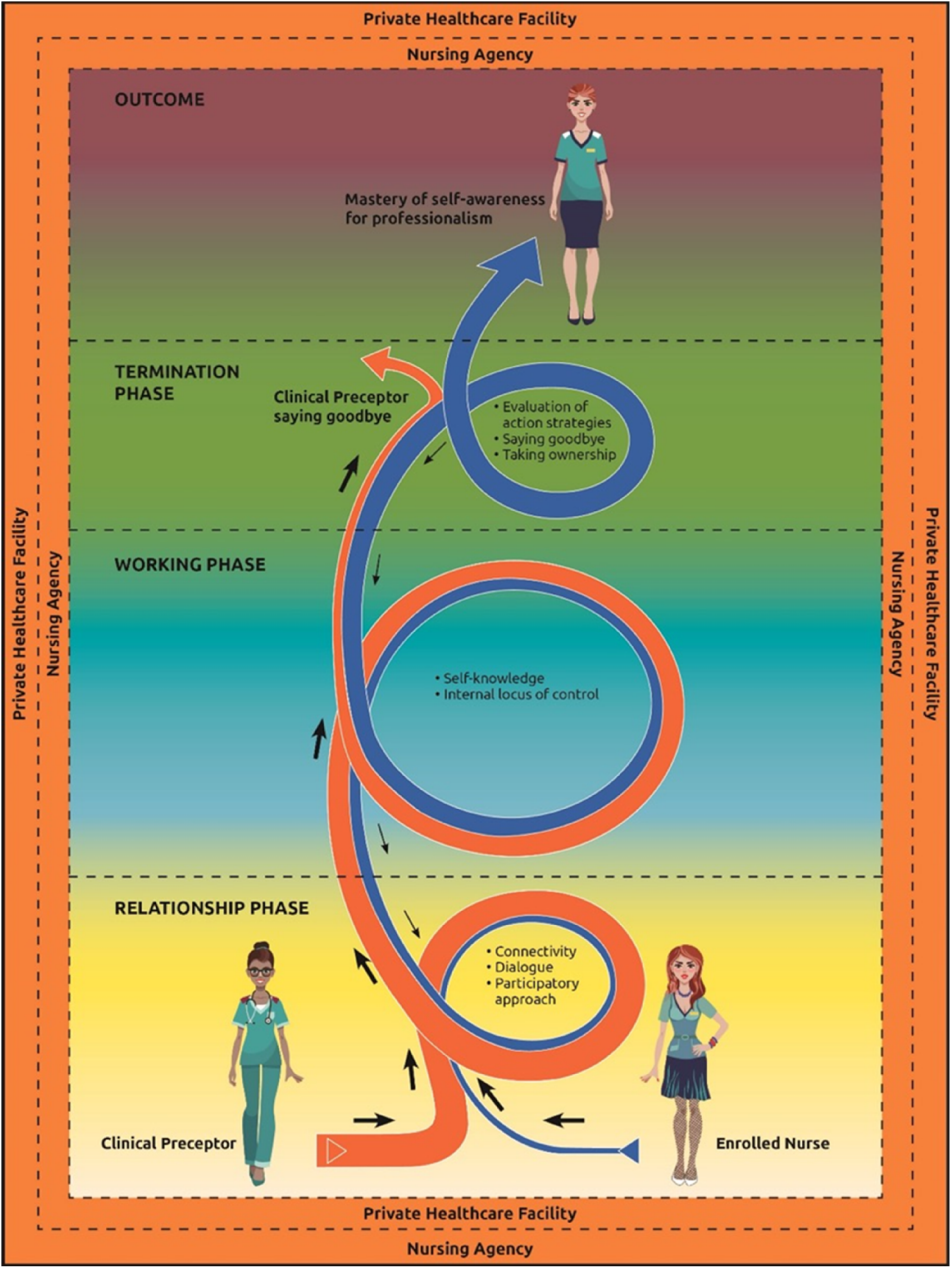
A model to facilitate self-awareness of professionalism, of enrolled nurses.

The clinical preceptor (participant) facilitates self-awareness of professionalism, for enrolled nurses, utilising three phases to reach an outcome. Figure 4 depicts the model to facilitate self-awareness for professionalism, of enrolled nurses. The model commences with the relationship phase when the enrolled nurse attends the nursing agency's orientation programme conducted by the clinical preceptor. The nursing agency offers a temporary employment service to support private healthcare facilities with supplemental nursing staff. The nursing agency is approached by all categories of nurses who are either unemployed and choose to work part-time, or nursing staff who are permanently employed in a specific private or public healthcare facility and choose to work in a different facility for supplemental income; also known as ‘moonlighting’. The enrolled nurses who choose to register at the nursing agency are required to attend an orientation programme. An orientation programme presents information about policies that regulate nursing agency staff, and information that the private healthcare facilities have requested the nursing agency relay to their staff before placement. During the nursing agency's orientation programme, the clinical preceptor commences with the model, namely the relationship phase.

The relationship phase is enabled through the clinical preceptor's mobilisation of resources. These resources include the invitation to dialogue (Severinsson, 2001), maintaining dialogue, and forming connectivity between the clinical preceptor and the enrolled nurse. The invitation to dialogue, maintain dialogue, and connectivity makes it easier for the enrolled nurse to participate in the facilitation process. At this stage of the relationship phase, the enrolled nurse is unaware of their professionalism. As part of the mobilisation of resources, the clinical preceptor operationalises the process of guiding the enrolled nurse, leading directly to the working phase.

The working phase focuses on the enrolled nurse's efforts to master self-awareness for professionalism. The working phase commences as the clinical preceptor distributes the Johari Model Worksheet (Luft, 1969). The Johari Model Worksheet is based on the Johari Model (Luft, 1969) that enables the user of the model to identify the disparity of what is known (quadrant one) to what is unknown by the self but known by others (quadrant two). The researcher focused on quadrant one and quadrant two of the Johari Model. This is the starting point for the enrolled nurse to become aware and attach meaning to self-knowledge and identify their internal locus of control for professionalism.

The last phase of the model is the termination phase. In this phase, there is an assessment of the enrolled nurse's mastery of self-awareness for professionalism. During this phase, the clinical receptor says goodbye as the enrolled nurse master's self-awareness for professionalism.

#### Structure of the Model

The structure of a model entails the model's purpose, the assumptions, conceptual definitions and a structural description (Chinn et al., 2022, pp. 160–169). The model's purpose, assumptions and central conceptual definition will be presented. The overall purpose of this model is specific to its context (Chinn et al., 2022, pp. 45–49, 160) and moreover to clinical practice, being nursing behaviours and nursing actions of enrolled nurses. Chinn, et al. (2018:181, 186), explain that assumptions are the basic truths accepted by the researcher. The assumptions of the model in this study are based on the Johari Model (Luft, 1969) and the Nursing Supervision Model (Severinsson, 2001). Once the assumptions were made, the model was understood in its own terms. Conceptual definitions were presented earlier, the central conceptual definition for the ‘facilitation of self-awareness for professionalism’ was defined as: ‘the enrolled nurse receiving guidance from the clinical preceptor through a process of mobilising resources for the enrolled nurse to attain self-knowledge and an internal locus of control for the competency to display the behaviour that is expected within the environment’.

#### Model's Evaluation

**Figure 5.**
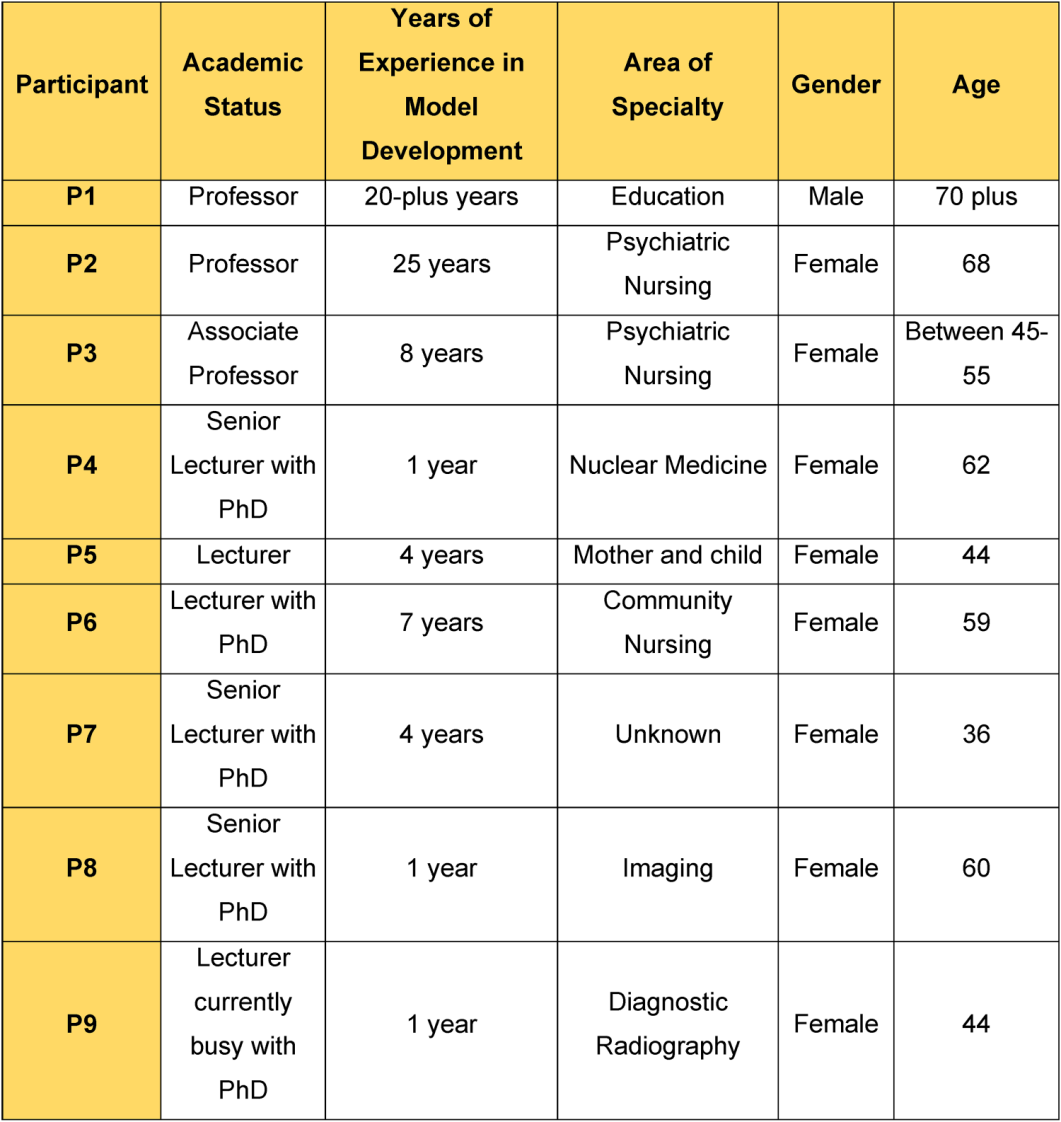
Profile of panel experts in the model evaluation.

The model was evaluated at a presentation by a panel of experts who were knowledgeable about model development. Figure 5 presents the profile of the panel of experts.

The panel evaluated the model by answering questions on the model's clarity, simplicity, generality, accessibility and importance (Chinn et al., 2022, pp. 169–176). Direct quotations from the panel experts are:

“Model is clear”

“Simple to follow. The colours are telling a story”

“General. It can be used in ALL private hosp and nursing ag (agencies)”

“Model is accessible and can be used in other contexts”

“Model is important for nursing professionalism and a guide to the ethical codes of the profession be it nursing, radiology, etc.”

The researcher read through all the panel expert comments for each question that was asked. Comments that suggested changes or removal of certain elements in the model were discussed with the researcher's supervisors and the changes were implemented. An example of a change pertained to the words in the model, namely ‘agent’ and ‘recipient’. An expert panel member suggested that these words be removed and the words ‘enrolled nurse and ‘clinical preceptor’ be added instead. See Figure 4.

Secondly, a recommendation was made by the panel member expert to change the study title change from ‘A Model to Facilitate Professional Socialisation of Enrolled Nurses at a Nursing Agency’ to ‘A model for the Facilitation of Self-Awareness for Professionalism of Enrolled Nurses at a Nursing Agency’. In a discussion with the researcher and supervisors it was decided that the new proposed title was more descriptive. A title change request was submitted and accepted by the Faculty of Health Sciences Higher Degrees Committee.

Lastly, the panel of experts concluded that the model is important to the field of nursing practice.

### Stage 4: Model Implementation and the Evaluation of the Model's Functionality

#### Step 1 Model Implementation

Prior to model implementation, the researcher made telephonic contact with several nursing agencies and introduced and presented the study through a physical meeting to the Chief Executive Officers and to the Senior Educational Managers. The researcher was left with one nursing agency who was willing to participate in the study. The nursing agencies that did not participate, provided telephonic reasons, one nursing agency explained that their clinical preceptor was too inundated with nursing staff support in patient care, due to the Covid-19 pandemic; and another nursing agency indicated that they were not willing to participate. This left the researcher with one nursing agency who was willing to participate in the study.

Model implementation was commenced through a one-day workshop held with the clinical preceptors, from the nursing agency. The co-ordination of the one-day workshop was arranged by the Head of Department (HOD) clinical preceptor because each clinical preceptor was assigned to various healthcare facilities, and the HOD clinical preceptor was able to arrange a specific date in which all the clinical preceptors could meet.

No sampling was done because all the participants were invited to the workshop. The nursing agency employed a total of seven clinical preceptors. Seven participants took part in the workshop. They were all registered nurses with varying years of experience in conducting orientation programmes.

The researcher presented the model ‘for the facilitation of self-awareness, for enrolled nurses’, to the clinical preceptors. The guidelines for the model's implementation were described in detail. The researcher invited the clinical preceptors to participate in the study and handed an information letter and a consent letter to each clinical preceptor. The information letter contained the researcher's contact details. The workshop was divided into three parts: namely, the introductory part, the operative part of the workshop and the concluding part of the workshop. The three parts of the workshop were specifically chosen by the researcher, to duplicate the structure of the model.

#### Step 2 Evaluation of the Model's Functionality

An evaluation of the model's implementation took place three months after its implementation. The researcher was telephonically informed that four clinical preceptors withdrew from the study due to the high demand for nursing staff to care for critically ill patients. Clinical preceptors became part of the workforce during the Covid-19 Delta variant, and some clinical preceptors had fallen ill and were emotionally and physically exhausted. Consequently, they could not implement the model.

#### Data Collection

Due to the increased demand for patient care during the Covid-19 pandemic (Delta variant), the clinical preceptors temporarily left their clinical preceptor post to nurse critically ill patients within private healthcare facilities, and some clinical preceptors were taken ill. This led to only one unstructured interview being conducted, two naïve sketches and two observational notes were received from the clinical preceptors.

Data collection began with an unstructured interview with a clinical preceptor (Participant 7). The interview was audio-recorded and conducted in English. The researcher asked the clinical preceptor the following question: **“How did this model work for you?”.** The interview was transcribed verbatim and analysed using thematic coding. The researcher used the primary source for thematic coding (Tesch, 2013, pp. 142–145) to maintain authenticity and credibility (Holloway & Galvin, 2017, pp. 119–120). Tesch's (2013:, pp. 142–145) thematic coding process was used to organise the data collected from the unstructured interview, the clinical preceptors’ naïve sketches, observational notes, the researcher's field notes and reflective notes.

At this point, after the researcher implemented Tesch's (2013:, pp. 142–145) coding process, the unstructured interview's transcribed data, the naïve sketches and observational notes from clinical preceptors were sent to an independent coder who analysed the data. During the time of this study, nursing shortages and patient overloads within hospitals, reached unprecedented times, patient care was an unquestionable priority (French et al., 2022, p. 45; Turale & Nantsupawat, 2021: 12). It was at this point that the researcher turned towards literature for guidance. Literature alludes to the point that a sample size cannot be detached from the study's characteristics that influence saturation (Hennink & Kaiser, 2022, p. 292). Furthermore, Braun and Clarke (2021, p. 205, 206) refer to saturation as *meaning* and that *meaning* is ‘in’ data. With this in mind, the researcher sought to find meaning in the data from the unstructured interview's transcribed data, the naïve sketches and observational notes. Braun and Clarke (2021, p. 206, 207) delineate the different types of thematic analyses, and refer to coding reliability, codebook and reflexive. In addition, Braun and Clarke (2021, p. 209) suggest that coding reliability measures that coders have been trained to code in the same way using codes designed to facilitate the measurement of coding agreement, however, when a reflexive thematic analysis is conducted, there is no consensus between coders, but rather that saturation comes from depth of engagement with data and in reflexive interpretation. The researcher adhered to Tesch's thematic analysis in identifying themes and thereafter, spent further time with the data to acquire meaning and a richness within the data and to ensure that the themes represent the preceptor's experiences.

The limited sample size was impacted by the staffing shortages in the nursing agency and the workload demands placed on nurses, including preceptors who took care of patients during the Covid-19 pandemic.

Prior to receiving communication from the independent coder, regarding the results, the researcher read through the transcribed data, the naïve sketches and observational notes from clinical preceptors in order to reflect and interpret the collected data.

**Figure 6.**
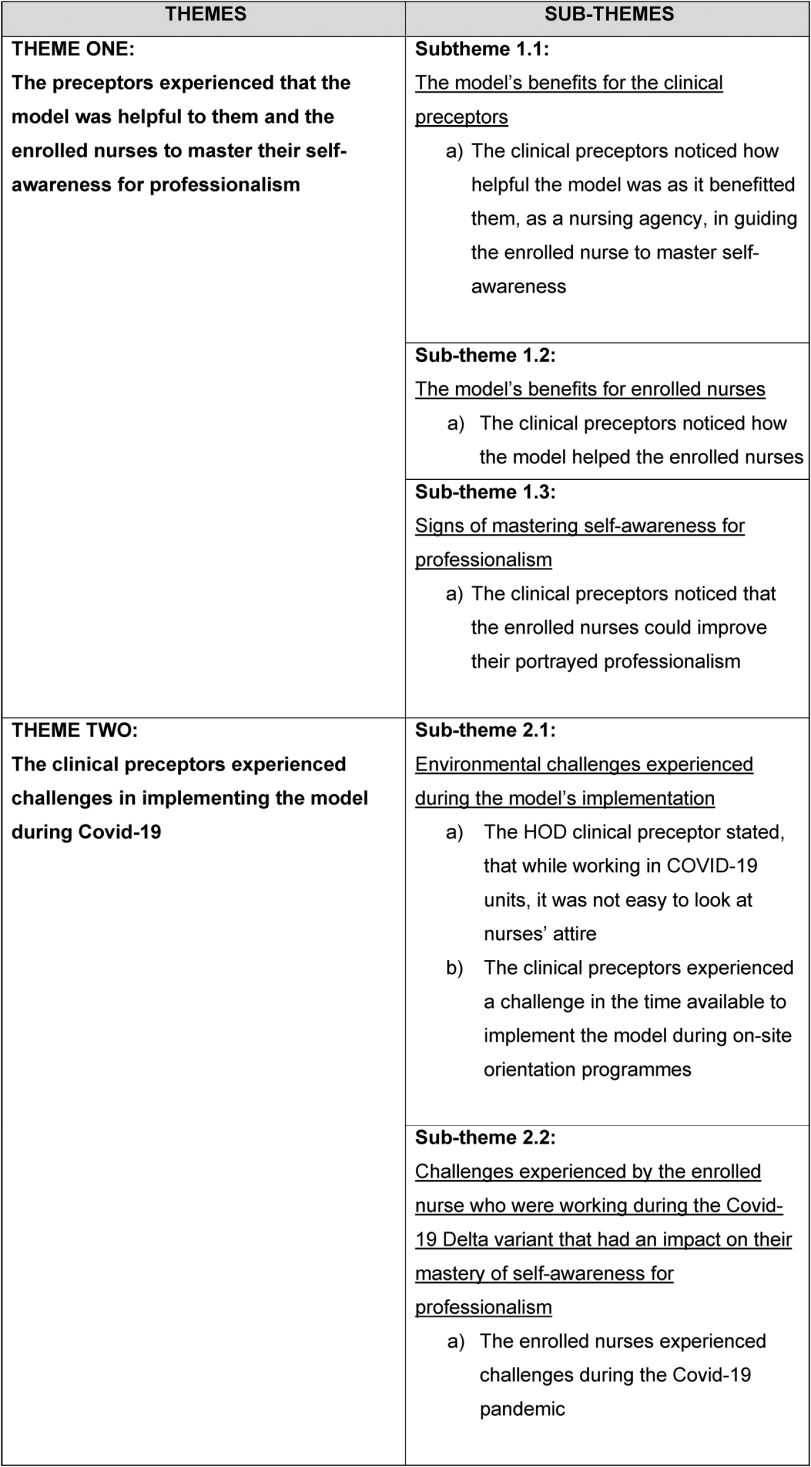
Consensus of themes and sub-themes.

The researcher and the independent coder met to discuss the themes and categories identified. The meeting took place using Microsoft Teams in which audio, video and screen sharing can be done. The independent coder shared her screen so that the researcher could see the themes and categories identified. The reason for the meeting was to discuss the themes and sub-themes identified by both the research her and the independent coder, thereby ensuring credibility in the truth of the data and in the interpretation of the data. A consensus was reached, confirming the themes and sub-themes. Figure 6 depicts the consensus of the themes and sub-themes.

## Results

Two themes were identified. Each theme contained a sub-theme/s. Each sub-theme is supported by direct quotations.

## Theme one: the Preceptors Experienced That the Model was Helpful to Them and the Enrolled Nurses to Master Their Self-Awareness for Professionalism

### Sub-Theme 1.1: The Model's Benefits for the Clinical Preceptors

a) The clinical preceptors noticed how helpful the model was as it benefitted them, as a nursing agency, in guiding the enrolled nurse to master self-awareness

“I can say that this helpful because [I] have seen a change” and “Yes, EN's saw their unacceptable behaviour problem …” “they are able to see for themselves the problems and make positive change for the agency” (Participant 2, Naïve sketch).

“The model is useful as it guided me to look at my behaviour and how I act, it helped me to introspect myself” (Participant 7, observational note).

“… it actually helped us to like, to enlighten us, to look more closely onto their behaviour” (Participant 7, unstructured interview).

“… and for like one or two of my Preceptors they found that this model helped them, not only identify the short comings of the EN but also of us, you know” (Participant 7, unstructured interview).

“Improvement noted”, “staff are tired but can see they trying”, “put name sticker on PPE” (Participant 4, observational note).

The clinical preceptors’ verbatim quotes indicate that they felt this model benefitted them, enabling and helping them to scrutinise the enrolled nurses’ professional behaviour. A skilled facilitator is able to identify shortfalls in their programmes and draws on knowledge to optimise the programme's effectiveness (Jones, 2021, pp. 4–5). The optimisation of a programme not only draws on the facilitator's knowledge but also adds and supports future decision-making (Chinn et al., 2022, p. 45). In addition, the clinical preceptors identified their own shortfalls enabling them to scrutinise their own processes thereby optimising their programme's effectiveness.

### Sub-Theme 1.2: the Model's Benefits for Enrolled Nurses

a) The clinical preceptors noticed how the model helped the enrolled nurses

The clinical preceptors stated that the model was helpful in supporting the enrolled nurses to master self-awareness for professionalism. They explained:

“We actually enjoyed running the model for you because it also … helped our staff members …” and “… it was self-reflected on them and it made them realise” (Participant 7, unstructured interview).

“Seem to understand / internalise. Better and improved.” and “… she was surprised, didn’t realise the impact on others” (Participant 4, observational note).

“… for the EN's who saw the difference between what others say and about them and what they see in terms of professionalism…were surprised!” (Participant 2, Naïve sketch).

The quotes indicate that the enrolled nurses noted the disparity in what was known to them and what was unknown to them but known to others. Recognising this disparity is the first step in gaining a mastery of self-awareness. It is necessary for essential behaviour (referring to what is known to others and not known to self) to be brought to light so that one can begin to deal with it (Luft, 1969, p. 14, 15; Woodward et al., 2019, pp. 54–57, 63–65). Within this study's context, the enrolled nurse must first be able to identify behaviour that is unknown to self but known to others; through the identification process, self-knowledge is gained.

### Sub-Theme 1.3: Signs of Mastering Self-Awareness for Professionalism

a) The clinical preceptors noticed that the enrolled nurses could improve their portrayed professionalism

The following quotations support the signs of enrolled nurses’ mastery of self-awareness:

“One EN felt her make-up is fine as a woman like other woman that are not nurses and feels confident when wearing make-up with her long hanging earrings” and “identified that there is part of make-up she can’t wear at work, may only apply when she is going out for some outing events, which sounded reasonably professional to me.” and “Yes, EN's saw their unacceptable behaviour problem and the effect on the profession…will try to correct it.” (Participant 2, Naïve sketch).

“… so it definitely the hairstyles changed they were neater, those that were growing their nails, the nails became shorter…” (Participant 7, unstructured interview).

“I can see the change in behaviour, cannot see uniform cos of PPE … more respectful and greeting.” (Participant 7, observational note).

The enrolled nurses’ mastery of self-awareness for professionalism in this study is based on the following three determining points:

1. The ability to identify the disparity between what is known to self and what is unknown to self but known to others.
2. The ability to regulate (control) their portrayed professionalism.
3. The ability to associate a causal relationship between their behaviour and the potential consequences/outcomes of the behaviour.

The ‘act of noticing’ is the beginning of mastering self-awareness, identifying what was once ‘unknown’ to known’. In the naïve sketch (above), the enrolled nurse realised her unprofessional portrayal and showed her ability to regulate her behaviour; that was to rather wear her “*make-up with her long hanging earrings”* when going for outings. The choice to regulate one's actions, displays an internal locus of control (Kesavayuth et al., 2020, pp. 227–238; Liang et al., 2019, p. 2). Furthermore, an internal locus of control implies that one can associate a causal relationship between one's behaviour and the potential consequences or outcomes (Peltokorpi et al., 2022, pp. 177–202; Kesavayuth et al., 2020, pp. 227–238).

## Theme Two: the Clinical Preceptors Experienced Challenges in Implementing the Model During Covid-19

### Sub-Theme 2.1: Environmental Challenges Experienced During the Model's Implementation

a) The HOD clinical preceptor stated, that while working in COVID-19 units, it was not easy to look at nurses’ attire

“And we had to don and doff, so we also had to wear scrubs, you know things like that, and uhm, and you know the patients were very sick and there were lots of tasks to be done, and I found the challenge was also to get them to give us their time” (Participant 7, unstructured interview).

“Unit busy”, “short [of] staff” and “staff running around, don’t have time to talk” and “too much to do, need to help staff on [the] floor” (Participant 7, observational note).

“Staff are wearing scrubs. Hair-caps and shoe covers used (worn)” and “Donning and doffing of PPE, staff come in own clothes – change into scrubs” and “can’t wear name badge – covered with PPE” (Participant 4, observational note).

“Not easy to see, all look the same when in PPE” and “One EN, he told me … [he] feels like he can collapse … doesn’t have energy – working ten days in a row. Saw another EN and started to talk to her but then went to the patient” (Participant 2, Naïve sketch).

During the Covid-19 pandemic, many adjustments were required within nursing care, including the donning and doffing of personal protective equipment (PPE) (Oliveira, 2020, pp. 1–2; Razu et al., 2021, pp. 1–2). This meant that nursing staff entering Covid-19 units’ attire looked similar, making it impossible to address professional attire. In addition, staffing shortages increased workload, limiting time for discussions that were not patient-related (Oliveira, 2020, p. 1, 2; Razu et al., 2021, p. 1).

b) The clinical preceptors experienced a challenge in the time available to implement the model during on-site orientation programmes

“Uh, it was very difficult running the, this, this model … normally what we do is, we use to have orientation in the classroom for five days before we take them to the field, but because of Covid and the shortage of staff we didn’t have that privilege, and because of the social distancing and the number of people we could train and couldn’t train. So, what we did was, we went straight into the, into the Units, and that's how we did it and uhm, with Covid we managed to do at least twenty EN's, and that was only on induction [orientation]” (Participant 7, unstructured interview).

“Time is too short, need to help with patients”, “… will run it at the agency” (Participant 7, observational note).

“…exercise took longer than expected… was interrupted frequent intervals…” and “we started but needed to attend to patient” and “… saw them during lunch time … 2 were interested and wanted to partake [participate] – others too exhausted” (Participant 4, observational note).

“Managed to implement model some of the time. But when short staff – not possible” and “Staff load heavy, many critical ill patients … must stretch” (Participant 2, Naïve sketch).

The clinical preceptors were challenged with limited time for model implementation due to workforce shortages occurring during the Covid-19 surges. Shun (2021:, p. 1) points out that the Covid-19 pandemic, together with nursing shortages, negatively impacted nurses’ available time for any activity that was not nursing care related. Literature emphasises the reality of nursing shortages impacted and increased the workload among healthcare workers (Oliveira, 2020, p. 1, 2; Razu et al., 2021, p. 1).

### Sub-Theme 2.2: Challenges Experienced by the Enrolled Nurses who Were Working During the Covid-19 Delta Variant That had an Impact on Their Mastery of Self-Awareness for Professionalism

a) The enrolled nurses experienced challenges during the Covid-19 pandemic

“… their working environment because they feel for patients you know they feel for the patients not able to see a visitor, for the patient dying alone, those are the things that came out in while using this model, you know it didn’t only limit them to how they look how they spoke whatever, personal feelings came out” (Participant 7, unstructured interview).

“The workload is sometimes too much and staff breakdown. To see patients like this and families outside is too much” and “I know these staff … they can manage the model and improve for professionalism but not now / Covid” (Participant 2, Naïve sketch).

“I saw one EN, she came to me to go through the model. [I] can see tears in her eyes. When asked what is wrong, she cries. She [EN] wants to do the model, she is a person who wants to develop herself but Covid is too much” (Participant 4, observational note).

No studies could be found that focus specifically on the enrolled nurses’ challenges during the Covid-19 pandemic. However, several studies have been conducted that explored nurses’ challenges overall during the Covid-19 pandemic. Among these challenges, psychological distress was a common theme (Oliveira, 2020; pp. 1–2; Razu et al., 2021, p. 1; Shun, 2021, p. 1). While and Clark's (2021:, pp. 384–388) study emphasise the ramifications of increased workload combined with environmental and social factors, affects one's mental wellbeing. The above quote “… *you know they feel for the patients … for the patient dying alone”* depicts the nurses’ emotions of caring for their patients, and furthermore that the enrolled nurses’ attention was placed on their patients before they turned their attention towards themselves. The above quote continues “… *those are the things that came out in while using this model”* further indicates their placement of attention, and hence impacted on their mastery of self-awareness.

## Conclusions

The purpose of this article was to describe the development and implementation of a model to facilitate self-awareness for professionalism for enrolled nurses. The article presents the background and unique contextual influences, that impact the nursing profession in South Africa. Chinn et al. (2022, pp. 148–157) four stages of model development is presented. The functionality of the model was evaluated by the clinical preceptors (who were the participants). It was determined that the model was functional. Findings indicated challenges faced by both the clinical preceptors and the enrolled nurses during the Covid-19 pandemic. the clinical preceptors experienced that the model was helpful to the enrolled nurses in guiding them to master self-awareness of professionalism. This was evident in the enrolled nurses’ ability to notice their portrayed professionalism, followed by an internal locus of control to improve their portrayed professionalism.

## Strengths and Limitations

Several limitations were identified in this study. The first was seen in the nursing shortages during the Covid-19 Delta variant in South Africa. Consequently, the clinical preceptors became part of the workforce to care for critically ill patients, affecting their participation in the study. This limited the sample size. Together with the impact of social distancing regulations, the remaining three clinical preceptors conducted the nursing agency's orientation programme, but an abbreviated version. Additionally, some of the clinical preceptors had taken ill and were unable to continue with the model's implementation. Covid-19 also affected the data collection phase in that the clinical preceptors were ill and were mentally and physically exhausted. Naïve sketches and observational notes were used to collect data in addition to the unstructured interview, however, it would have been more beneficial to conduct interviews with the two remaining clinical preceptors.

## Recommendations

The researcher recommends that the model be used in the domain of nursing practice and nursing research. The model is applicable as a frame of reference for nursing agencies, providing supplemental nursing personnel to private sectors. It is also applicable to public nursing sectors where portrayed professionalism necessitates enhancement.

The researcher recommends that this model be incorporated into the domain of nursing education because it relates to an important aspect of growth and maturity in one's career. That is to say, the facilitation of self-awareness for professionalism can advance one's career, or the lack thereof may impede one's career. The clinical preceptors experienced the model as valuable because they noticed enrolled nurses became aware of their portrayed unprofessionalism and identified an internal locus of control to make necessary changes in their behaviour. Additionally, the clinical preceptors tested the model on registered nurses (at the request of a unit manager), and positive results were found.

It is recommended that the model to facilitate self-awareness of professionalism for enrolled nurses be implemented at another nursing agency in with a larger sample size, within the South African nursing context.

Lastly, the researcher recommends that further research be conducted on the experiences of nurses who have participated in the model to explore the model's use as a tool for reflective practice.
